# FAPI-positron emission tomography for radiotherapy in head and neck cancer: applications and future directions

**DOI:** 10.1016/j.phro.2025.100792

**Published:** 2025-06-05

**Authors:** Mischa de Ridder, Remco de Bree, Bart de Keizer

**Affiliations:** a University Medical Center Utrecht, Department of Radiotherapy, Netherlands (the); b University Medical Center Utrecht, Department of Head and Neck Surgical Oncology, Netherlands (the); c University Medical Center Utrecht, Department of Radiology and Nuclear Medicine, Netherlands (the)

**Keywords:** Head and neck cancer, FAPI-PET, Radiotherapy, Response assessment

## Abstract

Radiotherapy is a cornerstone in the treatment of head and neck squamous cell carcinoma (HNSCC). Imaging plays an important role in target delineation in order to achieve adequate target dose and on the other hand limit radiation-induced toxicity to surrounding healthy tissues. Advancements in imaging techniques, including functional imaging with positron emission tomography (PET), have improved accurate target delineation, allowed for adaptive radiotherapy, and improved response evaluation.

This review explores the potential role of fibroblast activation protein inhibitor (FAPI)-PET/CT in radiotherapy planning for HNSCC, comparing its efficacy to the current standard, fluorine-18 fluorodeoxyglucose (18F-FDG) PET/CT.

FAPI-PET/CT is an emerging imaging modality that for pretreatment imaging may increase lesion detection, especially near the skull base. For target delineation FAPI PET/CT may enhance the specificity of tumor delineation, particularly in distinguishing tumor tissue from normal tissue. Current evidence suggests that FAPI-PET increases the GTV volume, but whether this represents more accurate tumor delineation or overestimation remains unclear. For response monitoring after treatment, there is no evidence in head and neck cancer, but for rectal cancer FAPI-PET/CT showed promising results in detection of residual disease after neo-adjuvant radiotherapy.

For now, FAPI-PET/CT could be used as additional imaging, and should not be used as replacement for other imaging modalities. There is an urgent need for prospective validation studies to determine the clinical impact of FAPI-PET on radiotherapy planning and response monitoring.

## 1 Introduction

Radiotherapy is one of the pillars in head and neck cancer treatment, and the majority of patients diagnosed with head and neck cancer undergo some form of ionizing radiation, either as primary treatment or postoperative treatment with curative or palliative intent. Although radiotherapy is used as organ preserving therapy, patients still suffer from the side effects of radiotherapy. For instance, xerostomia [1], dysphagia [2] and hypothyroidism [3], which are clearly related to radiation doses to healthy tissue, may occur. Radiotherapy techniques are evolving rapidly. Image-guided radiotherapy (IGRT) (including magnetic resonance guided radiotherapy (MRgRT) [4,5]) and proton therapy [6] are all developed to spare healthy tissue as much as possible. Currently, radiotherapy treatments are more personalized, faster, and more precise. For example, advances in imaging-guided adaptive radiotherapy have enabled online adaptation based on anatomical changes. Because of that, smaller margins can be taken into account, leading to reduced toxicity [7]. With all the new techniques, the precision in the delivery of the radiation has become that high, that target delineation has become one of the most important critical steps in the treatment. The option of imaging the target volume during treatment allows either online or offline adaptive radiotherapy. After treatment the tumor response need to be monitored as accurate as possible (i.e. is there residual tumor yes or no) to be able to offer patients salvage treatments. The reference standard for response assessment is histology, however imaging studies try to limit the need for biopsy [8].

Imaging plays a pivotal role in target delineation [9], adaptive radiotherapy [10] and response evaluation [11]. Next to conventional anatomical imaging, functional imaging is an important tool for these steps. Recent studies, such as those by Smits et al. and Subramaniam et al. [12,13], have demonstrated that multimodal (CT, PET and MRI) imaging significantly improves interobserver variability regarding tumor delineation. This reduced interobserver variability enhances treatment accuracy. The introduction of multimodal imaging has led to improvements in interobserver variability.

Positron emission tomography (PET) with fluorine-18 fluorodeoxy-d-glucose (^18^F-FDG) has proven its role in many oncological fields, also in head and neck cancer [14]. Especially, the almost perfect negative predicting value of ^18^F-FDG-PET/CT in head and neck squamous cell carcinoma (HNSCC) made it such a useful tool in clinical practice [15]. Nonetheless, the positive predictive value of ^18^F-FDG-PET/CT is limited due glucose metabolism in inflammatory or infectious tissue, which can lead to overestimation of the target tissue and unnecessary wide irradiation of the target volume with consequently unnecessary irradiation of healthy tissue with associated morbidity.

Despite advancements in imaging, accurate differentiation between tumor tissue and inflammation remains a major challenge. A significant portion of HNSCC consist of stroma, including fibroblasts. These carcinoma-associated-fibroblasts (CAFs) have been identified to be important in radiotherapy resistance and invasiveness [16]. These CAFs show overexpression of fibroblast activation protein (FAP). Visualization of CAFs can be done by a PET-tracer which act as FAP inhibitor (FAPI) [17]. Since, CAFs are associated with tumor biology and radiotherapy response there might be an important role for FAPI-PET/CT. Several FAPI compounds have been developed, including FAPI-04, FAPI-46, FAPI-74, and FAPI-2286. They may differ in biodistribution or specific binding capacities [18]. These compounds can be labeled with fluorine-18 (^18^F) or gallium-68 (^68^Ga). Recent studies have suggested that FAPI-PET/CT may outperform FDG-PET/CT in delineating tumor boundaries and differentiating malignant from inflammatory processes [19,20]. Moreover, its potential application in radiotherapy planning and response assessment now is an area of active investigation [21].

The clinical implementation of FAPI-PET/CT is still in its early stages. Limitations such as tracer availability, cost, and the need for larger clinical validation studies need to be addressed.

This review aims to evaluate the potential role of FAPI-PET/CT in radiotherapy for head and neck cancer, with a specific focus on its application in target delineation, treatment planning, and response monitoring. Additionally, we will compare FAPI-PET/CT to FDG-PET/CT and discuss whether its improved specificity can lead to better tumor delineation, reduced radiation exposure to normal tissues, and improved patient outcomes.

## 2 Pre-treatment imaging

Pre-treatment imaging in HNSCC is used to image local extent of the tumor (T-stage), appearance of lymph node metastases (N-stage) and possible distant metastases (M-stage). Next to the diagnostic purpose, pre-treatment imaging is also used for radiotherapy planning. Accurate delineation of the primary tumor and metastatic sites is essential for optimizing treatment strategies.

### 2.1 Time between injection and image acquisition

According to the SNMMI guidelines [18] the uptake time of ^68^Ga-FAPI is between 30 and 60 min. Lesion uptake and detection has been reported stable with scanning times between 20 and 120 min postinjection for ^68^Ga-FAPI [18]. For ^18^F-FAPI the recommended scanning time postinjection is slightly tighter between 30 and 90 min postinjection [18].

The possible benefit of late scanning is that it may improve discrimination between tumor and inflammatory responses [22]. For HNSCC specifically a study was performed with 69 patients [23], scheduled for curative treatment. The patients underwent two ^68^Ga-FAPI PET/CTs, one 30 min postinjection and a delayed scan 2 h postinjection. This study found superior tumor to background ratio (TBR) for the delayed scans. Also, the discrimination between metastatic and non-metastatic lymph nodes was improved by delayed scanning represented by an increased detection rate (68 % versus 76 %) correlated to histopathologic examination of neck dissection specimens.

### 2.2 Lesion detection

A small study from Wegen et al. compared the diagnostic value of ^18^F-FDG-PET/CT and ^68^Ga-FAPI-PET/CT in 15 head and neck cancer patients scheduled for radiotherapy [21]. On average 147 MBq [^68^Ga]Ga-FAPI was administered, time between injection and image acquisition was not reported. They found that all patients had FAPI uptake in the primary lesion, with a significantly higher tumor-to-background ratio (TBR), defined as SUVmax in the lesion divided by SUVmean of the liver, in FAPI-PET/CT compared to FDG-PET/CT. In these 15 patients a total of 49 lesions were detected. Compared to conventional imaging, 5 patients were upstaged following PET imaging. There was no difference in these 5 patients between FDG and FAPI. Of the 49 detected lesions, 40 (82 %) were FDG-positive and 41 (84 %) were FAPI-positive. Discrepancies were found in 9 lesions, 4 of those were FAPI-negative/FDG-positive and 5 were FAPI-positive and FDG-negative. These differences suggest that FAPI-PET indeed reflects tumor-associated fibroblast activity rather than pure metabolic activity, which could influence target recognition and delineation in radiotherapy. The authors recommend using both tracers to obtain comprehensive diagnostic information.

They also delineated the lesions on conventional imaging (CT + MRI), FDG-PET/CT and FAPI-PET/CT. The volume of GTV delineations on PET/CT was significantly larger compared to GTV delineations on conventional imaging (median increase of 9.2 ml and 7.2 ml respectively). In none of the cases the PET bases delineations were smaller.

The study from Jiang et al. evaluated prospectively the diagnostic accuracy of ^68^Ga-FAPI PET/CT in 77 patients with head and neck cancer and compared it to FDG-PET/CT [24]. The intravenous injection dose of ^68^Ga-FAPI (1.85–2.96 MBq/kg) was determined according to the patient’s body weight and the PET/CT scan was performed 30–60 min post-injection. Sixty-seven of those were patients with primary tumors. All primary tumors were detected with both FAPI- and ^18^F-FDG-PET/CT. In this study there was no difference in TBR (defined by SUVmax of the lesion divided by SUVmean of normal contralateral tissue) between FAPI- and FDG-PET/CT (9.78 ± 6.16 vs 9.31 ± 5.61, p = 0.525). The tumor to blood pool (TBPR), defined by SUVmax of the lesion divided by SUV mean of the blood pool was significantly greater for FAPI-PET/CT (13.89 ± 6.98) compared to FDG-PET/CT (8.37 ± 4.29, p = 0.000). Also, FAPI-PET revealed skull base and intracranial extension in 4 patients, whereas FDG-PET/CT failed to detect these due to high physiological FDG uptake in the brain. Twenty-nine of the patients with a primary tumor underwent surgical treatment with neck dissection, in total 40 neck sides with 906 lymph nodes. Of these, 21 patients had lymph node metastases, with a total positive lymph nodes of 78. The sensitivity for detection of positive lymph nodes was comparable between FAPI and FDG (85.7 % vs 85.7 %, p = 1.000), however the specificity was significantly higher for FAPI compared to FDG (87.5 % vs 12.5 %, p = 0.031) and also the accuracy was higher (86.2 % vs 65.5 %, p = 0.070). ^68^Ga-FAPI showed considerably higher diagnostic accuracy than ^18^F-FDG PET/CT in evaluating the N0 and N1 neck status (80.0 % (12/15) vs 20.0 % (3/15), p = 0.004). The reference standard in these analyses was histology and/or other imaging.

Another head-to-head comparison was done in 15 patients with nasopharyngeal carcinoma. All patients were scanned with ^68^Ga-FAPI PET-CT and ^18^F-FDG PET/CT. The ^68^Ga-FAPI was administered 30–60 min before image acquisition. Again, both PET-CTs had a detection rate of the primary tumor of 100 %. The T-stage on ^68^Ga-FAPI-PET/CT was consistent with MRI, whereas the T-stage was underestimated on ^18^F-FDG PET/CT in 1/15 patients. Especially, the skull base extension was comparable between MRI and ^68^Ga-FAPI PET/CT. Both MRI and ^68^Ga-FAPI PET/CT outperformed ^18^F-FDG PET/CT in visualizing skull base extension in all 8 patients with such extension. The same holds for intracranial extension. Four patients in this study had intracranial extension that was missed on ^18^F-FDG-PET/CT. ^68^Ga-FAPI aided the detection of three small skull lesions in two patients, which were initially overlooked during MRI interpretation and false-negative on the ^18^F-FDG-PET images.

For patients with head and neck cancer of unknown primary (HNCUP), PET imaging plays a crucial role in identifying occult tumors. FDG-PET/CT is commonly used in this setting, but its sensitivity is limited by physiological FDG uptake in the oropharynx and base of the tongue, leading to challenges in primary tumor localization. In case the primary tumor location cannot be found with imaging and/or examination under general anesthesia most centers tend to treat the entire pharyngeal mucosa (in case HPV/EBV negative disease) or the entire nasopharynx (in case EBV positive) or the entire oropharynx (in case HPV positive). This increases the amount of normal tissue being irradiated. If the primary tumor can be detected the radiation field can be reduced substantially from the whole oropharyngeal mucosal lining (Human Papilloma Virus (HPV)+) or pharyngeal and laryngeal mucosal lining (HPV−) to the (small) primary tumor location. Since the primary HPV-positive oropharyngeal carcinoma is often small (and therefore occult) and physiological FDG-uptake may hamper detection of occult oropharyngeal cancer, particularly HPV-positive HNCUP may benefit from more tumor specific tracers like FAPI. The incidence of HPV-positive oropharynx cancer is increasing extremely.

Emerging evidence suggests that FAPI-PET/CT may improve detection of unknown primary tumors, particularly in cases where FDG-PET fails [25,26]. The first study by Gu et al. investigated the use of ^68^Ga-FAPI PET/CT in 18 HNCUP patients with negative FDG-PET/CT findings. FAPI-PET/CT successfully identified the primary tumor in 38.9 % of these cases, localizing tumors in the nasopharynx, palatine tonsil, submandibular gland, and hypopharynx [25]. The second study by Gu et al. prospectively evaluated the benefit of FAPI-PET/CT and FDG-PET/CT in patients with cervical squamous cell carcinoma lymph node metastases and negative findings on clinical examination and imaging (CT and MRI) for primary tumors. The found that FAPI-PET/CT detected significantly more primary tumors (46 versus 17, p < 0.001) compared to FDG-PET/CT. This led to treatment changes in 22 out of 91 patients (24 %). A comparative summary of these studies is provided in Table 1, highlighting differences in sensitivity, specificity, and tumor detection rates.

**Table 1:** Summary of comparative studies evaluating FDG-PET/CT and FAPI-PET/CT for head and neck cancer. Sensitivity and tumor specificity were assessed for primary tumors, lymph node metastases, and unknown primary tumors. FAPI-PET/CT demonstrated higher specificity and lesion detection accuracy compared to FDG-PET/CT, particularly in cases of skull base invasion and unknown primary tumors. References correspond to the cited studies in the manuscript.

Study	Number of patients	Indication	Tumor type	Golden standard	FDG sensitivity	FDG tumor specificity	FAPI sensitivity	FAPI tumor specificity
Wegen et al. (2022)	15	HNSCC radiotherapy planning	Primary tumor + lymph node metastases	Histopathology + MRI/CT	82 % (40/49 lesions)	Limited due to inflammation	84 % (41/49 lesions)	Higher than FDG, reduced false positives
Jiang et al. (2023)	77	Head and neck squamous cell carcinoma (HNSCC)	Primary tumor + lymph node metastases	Histopathology + MRI	85.7 %	12.5 %	85.7 %	87.5 %
Gu et al. (2022)	18	Head and neck cancer of unknown primary (HNCUP)	Unknown primary tumor detection	Histopathology + FDG-PET/CT	N/A (all FDG-negative cases)	Limited due to false negatives	38.9 % (Identified primary tumors in FDG-negative cases)	Improved detection of unknown primaries
Syed et al. (2020)	14	Head and neck tumor delineation	Primary tumor delineation	Clinical correlation + MRI	Not Reported	Not Reported	Higher tumor-to-background contrast than FDG	Higher than FDG for tumor margins
Röhrich et al. (2021)	12	Adenoid cystic carcinoma staging and contouring	Primary tumor staging + contouring	Histopathology + MRI/CT	Not Reported	Not Reported	Improved lesion detection compared to MRI	Better delineation of perineural spread
Gu et al. (2024)	91	Head and neck cancer of unknown primary (HNCUP)	Unknown primary tumor detection	Histopathology	25 %	Not reported	51 %	Not reported

These findings suggest that FAPI-PET/CT could play an important role in radiotherapy planning for HNCUP by allowing for more targeted irradiation of the primary tumor, potentially reducing toxicity associated with larger radiation fields.

Two recent *meta*-analyses on the comparison of FDG-PET/CT and FAPI-PET/CT showed that most studies suggest that 68 Ga-FAPI-PET/CT outperforms 18F-FDG-PET/CT in terms of specificity, tumor delineation, and lesion detection, particularly in cases of skull base invasion and unknown primary tumors [27,28]. However, these findings are based on small sample sizes, and prospective, large-scale trials are needed to confirm its clinical utility in diagnostic workflows and radiotherapy planning.

### 2.3 Tumor delineation

In HNSCC, where tumor boundaries can be obscured by adjacent inflamed or fibrotic tissues, FAPI PET offers the potential advantage of more precise tumor volume definition because of the high tumor-to-background ratio of FAPI PET [29]. As mentioned above, accurate high contrast imaging is particularly relevant for radiotherapy planning, as accurate delineation of the gross tumor volume (GTV) and clinical target volume (CTV) is essential for maximizing tumor control while minimizing toxicity to adjacent critical structures.

One of the first studies evaluating the use of FAPI PET/CT to detect and delineate head and neck cancer was the study by Syed et al. [30]. They included 14 head and neck cancer patients and compared FAPI PET/CT to conventional CT and/or MRI based GTV delineation. The images were acquired 30 min postinjection. FAPI GTV was defined by a three-, five-, seven- or tenfold increase of FAPI SUVmax in the tumor compared to normal tissue. The volumes of GTV differed significantly between conventional GTV and the four FAPI thresholds. When merged with conventional GTV and related to dose coverage, it was found that some regions of FAPI-positive tissue was not included in the conventional treatment plan. Outcome data was not available for this study.

Another study in patients with adenoid cystic carcinoma, used FAPI PET for staging, but also for auto-contouring purposes [31]. In this study patients were scanned 1 h post-injection. They found that in 5 out of 12 patients, additional tumor manifestations were detected, that were missed on contrast enhanced CT and/or contrast enhanced MRI. Four of these 5 patients had distant metastases in lungs or bones, while one patient had suspicious tracer uptake in the contralateral maxillary sinus. Since this was compared to conventional imaging it is unknown whether FDG-PET/CT would have captured these as well. For contouring purposes, the study compared 10 min, 1-h and 3-h postinjection imaging to CT and/or MRI. The 1-h postinjection images were considered the best, since 10 min postinjection had considerable amount of non-specific FAPI uptake and 3 h postinjection suffered from too much washout. The volumes between the contours based on conventional imaging or 3 time points FAPI PET did not significantly differ. In this study no overlay analyses (DICE/Hausdorff distances) were performed. Expert consensus revealed that FAPI GTVs were considered more accurate, since small perineural growth or branches were better covered with FAPI GTVs. Table 1 presents an overview of key studies evaluating tumor delineation with FAPI-PET/CT and FDG-PET/CT, supporting its potential role in radiotherapy planning.

Geographical misses are important quality metrics in radiotherapy. Especially, since the introduction of more conformal treatments the delineation has become one of the weakest links. A recent *meta*-analysis of 56 studies showed that the majority of local failures are within the high dose CTV (84 %) [32]. A bit worrisome is that they also showed that there is a trend observed towards more non-high dose failures in patients treated in more recent years. Meaning that geographical misses are a problem with modern radiation technique. In esophageal cancer it was observed that FAPI-PET/CT based delineations reduced geographical misses compared to FDG-PET/CT based delineations [33]. Again, this support the added value of using both tracers instead of one.

## 3 Response assessment

Currently, no studies have been published on the use of FAPI-PET/CT for response assessment in HNSCC, highlighting a significant gap in knowledge. However, insights from MRI and FDG-PET/CT demonstrate the challenges of response evaluation [34]. Especially, discriminating post-therapeutic changes from residual tumor.

MRI is commonly used post-treatment, particularly for differentiating tumor recurrence from post-radiotherapy fibrosis, with techniques such as diffusion-weighted imaging (DWI) and dynamic contrast-enhanced (DCE) MRI [35]. To uniform post treatment analysis of imaging multiple classification systems has been introduced and validated. Currently, the most used classification system is the NI-RADS [36]. However, FDG uptake interpretation within NI-RADS remains somewhat subjective, and additional imaging modalities could further refine response assessment [37].

FAPI-PET/CT, which targets cancer-associated fibroblasts rather than glucose metabolism, may provide a more specific marker for viable tumor tissue, potentially reducing false positives caused by inflammation. Additionally, FAPI uptake may offer insights into the tumor microenvironment and treatment resistance [38].

Supporting this concept, a study in rectal cancer patients treated with neo-adjuvant radiotherapy followed by surgery compared ^68^Ga-FAPI-PET/MRI, ^18^F-FDG PET/CT, and contrast-enhanced MRI to predict complete pathological response was performed. PET parameters, including standardized uptake value (SUV), metabolic tumor volume (MTV), and FAP-expressing tumor volume (FTV), were evaluated for diagnostic accuracy. Among FAPI-PET parameters, ΔSULpeak% had the largest area under the curve (AUC 0.929), with 77.78 % sensitivity and 100 % specificity. Although conducted in rectal cancer, these findings suggest that FAPI-PET could serve as a valuable response assessment tool in other malignancies, including HNSCC, warranting further investigation.

There are no prospective studies in HNSCC, so the role of FAPI-PET/CT in response evaluation remains unproven. Future research should focus on longitudinal studies comparing FAPI-PET/CT with MRI and FDG-PET/CT to assess its clinical value.

## 4 Future perspectives

FAPI-PET/CT imaging is emerging as a promising tool for head and neck cancer imaging, with potential applications in pretreatment imaging. For treatment response monitoring the role is unclear, since up till now no specific head and neck cancer studies are published on this topic. The study performed in rectal cancer shows promising results, so future studies in head and neck are needed and should be performed to show the role. Nonetheless, significant gaps in knowledge remain, particularly regarding the accuracy and clinical significance (i.e. vital tumor or not) of FAPI uptake. The studies discussed above consistently demonstrate that FAPI-PET/CT increases the GTV volume compared to conventional imaging. The critical question remains: Does this reflect an underestimation by conventional imaging, or is FAPI-PET/CT overestimating tumor extent? Outcome data from the study by Syed et al. [30] are expected and will show us in this small cohort what the meaning is of FAPI positive areas outside the GTV delineations. Will the use of FAPI-PET/CT in radiotherapy planning result in higher tumor control rates? To answer this more in depth, more robust data are essential. Future research should focus on:

• Comparing FAPI-PET/CT with histopathology to determine whether the additional tumor volume identified represents viable cancer cells or reactive fibroblast activity.
• Validating the role of FAPI-PET in radiotherapy planning by assessing its impact on treatment adaptation, toxicity reduction, and oncologic outcomes.
• Longitudinal studies on response assessment to evaluate whether FAPI-PET/CT improves post-treatment surveillance and the early detection of recurrence.

Preferably, robust data should be derived from well-designed prospective studies. Nonetheless, physicians could use FAPI-PET/CT (depending on local availability and reimbursement) already. Especially, the role in radiotherapy planning could be derived from real-world data.

In conclusion, FAPI-PET/CT holds considerable potential as an adjunct imaging modality in head and neck cancer, particularly in enhancing tumor delineation and identifying challenging sites such as skull base invasion or unknown primary tumors. Preliminary evidence indicates that FAPI-guided imaging often expands GTVs compared to conventional modalities, though it remains unclear whether this reflects improved tumor capture or overestimation.

At this stage, FAPI-PET/CT should be viewed as a complementary tool rather than a replacement for established imaging techniques. Its integration into radiotherapy planning may offer added value, especially in anatomically complex or diagnostically ambiguous cases. To establish its role in clinical practice, prospective studies are needed to validate its impact on treatment accuracy and patient outcomes.

## CRediT authorship contribution statement

**Mischa de Ridder:** Conceptualization, Investigation, Writing – original draft. **Remco de Bree:** Conceptualization, Writing – review & editing. **Bart de Keizer:** Conceptualization, Writing – review & editing.

## Declaration of competing interest

The authors declare that they have no known competing financial interests or personal relationships that could have appeared to influence the work reported in this paper.

## References

[1] Dijkema T., Raaijmakers C.P., Ten Haken R.K., Roesink J.M., Braam P.M., Houweling A.C. (2010). Parotid gland function after radiotherapy: the combined michigan and utrecht experience. Int J Radiat Oncol Biol Phys.

[2] Head M.D.A., Neck Cancer Symptom Working G. (2016). Beyond mean pharyngeal constrictor dose for beam path toxicity in non-target swallowing muscles: dose-volume correlates of chronic radiation-associated dysphagia (RAD) after oropharyngeal intensity modulated radiotherapy. Radiother Oncol.

[3] Chow J.C.H., Lui J.C.F., Cheung K.M., Tam A.H.P., Lam M.H.C., Yuen T.Y.S. (2022). Post-radiation primary hypothyroidism in patients with head and neck cancer: external validation of thyroid gland dose-volume constraints with long-term endocrine outcomes. Radiother Oncol.

[4] Mulder S.L., Heukelom J., McDonald B.A., Van Dijk L., Wahid K.A., Sanders K. (2022). MR-guided adaptive radiotherapy for OAR sparing in head and neck cancers. Cancers (Basel).

[5] Reinders F.C.J., de Ridder M., Doornaert P.A.H., C P.J.R., Philippens M.E.P. (2023). Individual elective lymph node irradiation for the reduction of complications in head and neck cancer patients (iNode): a phase-I feasibility trial protocol. Clin Transl Radiat Oncol.

[6] Tambas M., Steenbakkers R., van der Laan H.P., Wolters A.M., Kierkels R.G.J., Scandurra D. (2020). First experience with model-based selection of head and neck cancer patients for proton therapy. Radiother Oncol.

[7] Al-Mamgani A., Kessels R., Janssen T., Navran A., van Beek S., Carbaat C. (2022). The dosimetric and clinical advantages of the GTV-CTV-PTV margins reduction by 6 mm in head and neck squamous cell carcinoma: significant acute and late toxicity reduction. Radiother Oncol.

[8] de Ridder M., Gouw Z.A.R., Navran A., Hamming-Vrieze O., Jasperse B., van den Brekel M.W.M. (2019). FDG-PET/CT improves detection of residual disease and reduces the need for examination under anaesthesia in oropharyngeal cancer patients treated with (chemo-)radiation. Eur Arch Otorhinolaryngol.

[9] Gregoire V., Evans M., Le Q.T., Bourhis J., Budach V., Chen A. (2018). Delineation of the primary tumour Clinical Target Volumes (CTV-P) in laryngeal, hypopharyngeal, oropharyngeal and oral cavity squamous cell carcinoma: AIRO, CACA, DAHANCA, EORTC, GEORCC, GORTEC, HKNPCSG, HNCIG, IAG-KHT, LPRHHT, NCIC CTG, NCRI, NRG oncology, PHNS, SBRT, SOMERA, SRO, SSHNO, TROG consensus guidelines. Radiother Oncol.

[10] Nuyts S., Bollen H., Eisbruch A., Strojan P., Mendenhall W.M., Ng S.P. (2024). Adaptive radiotherapy for head and neck cancer: pitfalls and possibilities from the radiation oncologist's point of view. Cancer Med.

[11] Machiels J.P., Rene Leemans C., Golusinski W., Grau C., Licitra L., Gregoire V. (2020). Squamous cell carcinoma of the oral cavity, larynx, oropharynx and hypopharynx: EHNS-ESMO-ESTRO clinical practice guidelines for diagnosis, treatment and follow-up. Ann Oncol.

[12] Subramaniam R.M., Duan F.M., Romanoff J., Yu J.Q., Bartel T., Dehdashti F. (2022). (18)F-FDG PET/CT staging of head and neck cancer: interobserver agreement and accuracy-results from multicenter ACRIN 6685 clinical trial. J Nucl Med.

[13] Smits H.J.G., Raaijmakers C.P.J., de Ridder M., Gouw Z.A.R., Doornaert P.A.H., Pameijer F.A. (2024). Improved delineation with diffusion weighted imaging for laryngeal and hypopharyngeal tumors validated with pathology. Radiother Oncol.

[14] Caldarella C., De Risi M., Massaccesi M., Micciche F., Bussu F., Galli J. (2024). Role of (18)F-FDG PET/CT in head and neck squamous cell carcinoma: current evidence and innovative applications. Cancers (Basel).

[15] Gupta T., Master Z., Kannan S., Agarwal J.P., Ghsoh-Laskar S., Rangarajan V. (2011). Diagnostic performance of post-treatment FDG PET or FDG PET/CT imaging in head and neck cancer: a systematic review and meta-analysis. Eur J Nucl Med Mol Imaging.

[16] Wang Z., Tang Y., Tan Y., Wei Q., Yu W. (2019). Cancer-associated fibroblasts in radiotherapy: challenges and new opportunities. Cell Commun Signal.

[17] Park J.E., Lenter M.C., Zimmermann R.N., Garin-Chesa P., Old L.J., Rettig W.J. (1999). Fibroblast activation protein, a dual specificity serine protease expressed in reactive human tumor stromal fibroblasts. J Biol Chem.

[18] Hope T.A., Calais J., Goenka A.H., Haberkorn U., Konijnenberg M., McConathy J. (2025). SNMMI procedure standard/EANM practice guideline for fibroblast activation protein (FAP) PET. J Nucl Med.

[19] Ji M., Ma G., Liu C., Gu B., Du X., Ou X. (2024). Head-to-head comparison of [(68)Ga]Ga-DOTA-FAPI-04 and [(18)F]FDG PET/CT for the evaluation of tonsil cancer and lymph node metastases: a single-centre retrospective study. Cancer Imaging.

[20] Guglielmo P., Alongi P., Baratto L., Abenavoli E., Buschiazzo A., Celesti G. (2023). Head-to-head comparison of FDG and radiolabeled FAPI PET: a systematic review of the literature. Life (Basel).

[21] Wegen S., van Heek L., Linde P., Claus K., Akuamoa-Boateng D., Baues C. (2022). Head-to-head comparison of [(68) Ga]Ga-FAPI-46-PET/CT and [(18)F]F-FDG-PET/CT for radiotherapy planning in head and neck cancer. Mol Imaging Biol.

[22] Rohrich M., Naumann P., Giesel F.L., Choyke P.L., Staudinger F., Wefers A. (2021). Impact of (68)Ga-FAPI PET/CT imaging on the therapeutic management of primary and recurrent pancreatic ductal adenocarcinomas. J Nucl Med.

[23] Jiang Y., Huang S., Tian Y., Xing D., Xiao Z., Huang J. (2025). Dual-time point 68 ga-FAPI-04 PET/CT improves tumor delineation and cervical lymph node metastasis identification in patients with head and neck squamous cell carcinoma. Clin Nucl Med.

[24] Jiang Y., Wen B., Li C., Tian Y., Xiao Z., Xu K. (2023). The performance of (68)Ga-FAPI-04 PET/CT in head and neck squamous cell carcinoma: a prospective comparison with (18)F-FDG PET/CT. Eur J Nucl Med Mol Imaging.

[25] Gu B., Xu X., Zhang J., Ou X., Xia Z., Guan Q. (2022). The added value of (68)Ga-FAPI PET/CT in patients with head and neck cancer of unknown primary with (18)F-FDG-negative findings. J Nucl Med.

[26] Gu B., Yang Z., Du X., Xu X., Ou X., Xia Z. (2024). Imaging of tumor stroma using (68)Ga-FAPI PET/CT to improve diagnostic accuracy of primary tumors in head and neck cancer of unknown primary: a comparative imaging trial. J Nucl Med.

[27] Ma J., Zhang J., Zhao T., Deng J., Zhang C. (2025). Diagnostic value of (18)F-FDG and (68)Ga-FAPI in head and neck cancers: a systematic review and meta-analysis. Hell J Nucl Med.

[28] Huang S., Wang Y., Huang R. (2025). Comparison of (18)F-fluorodeoxyglucose PET and (68)Ga-fibroblast activation protein inhibitor PET in head and neck cancers: a systematic review and meta-analysis. Acad Radiol.

[29] Loktev A., Lindner T., Mier W., Debus J., Altmann A., Jager D. (2018). A tumor-imaging method targeting cancer-associated fibroblasts. J Nucl Med.

[30] Syed M., Flechsig P., Liermann J., Windisch P., Staudinger F., Akbaba S. (2020). Fibroblast activation protein inhibitor (FAPI) PET for diagnostics and advanced targeted radiotherapy in head and neck cancers. Eur J Nucl Med Mol Imaging.

[31] Röhrich M., Syed M., Liew D.P., Giesel F.L., Liermann J., Choyke P.L. (2021). (68)Ga-FAPI-PET/CT improves diagnostic staging and radiotherapy planning of adenoid cystic carcinomas – imaging analysis and histological validation. Radiother Oncol.

[32] Kristensen M.H., Nielsen S.B., Alsner J., Holm A.I.S., Hansen C.R., Overgaard J. (2025). A systematic review and proportional meta-analysis of image-based pattern of loco-regional failure analyses outcomes in head and neck squamous cell carcinoma. Radiother Oncol.

[33] Zhao L., Chen S., Lin L., Sun L., Wu H., Lin Q. (2020). [(68)Ga]Ga-DOTA-FAPI-04 improves tumor staging and monitors early response to chemoradiotherapy in a patient with esophageal cancer. Eur J Nucl Med Mol Imaging.

[34] Cheung P.K., Chin R.Y., Eslick G.D. (2016). Detecting residual/recurrent head neck squamous cell carcinomas using PET or PET/CT: systematic review and meta-analysis. Otolaryngol Head Neck Surg.

[35] Noij D.P., Martens R.M., Koopman T., Hoekstra O.S., Comans E.F.I., Zwezerijnen B. (2018). Use of diffusion-weighted imaging and (18)F-fluorodeoxyglucose positron emission tomography combined with computed tomography in the response assessment for (chemo)radiotherapy in head and neck squamous cell carcinoma. Clin Oncol (R Coll Radiol).

[36] Strauss S.B., Aiken A.H., Lantos J.E., Phillips C.D. (2021). Best practices: application of NI-RADS for posttreatment surveillance imaging of head and neck cancer. AJR Am J Roentgenol.

[37] Mishra A., Tewari V., Shukla S., Singh S.N., Shukla V., Sarkar S. (2024). NIRADS-based case assessment of post-treatment head and neck cancer and its clinical correlation: a validation study. Natl J Maxillofac Surg.

[38] Liu T., Han C., Wang S., Fang P., Ma Z., Xu L. (2019). Cancer-associated fibroblasts: an emerging target of anti-cancer immunotherapy. J Hematol Oncol.

